# Focus on comfort: the effect of focus language on postoperative hospital stay compared to the Numeric Rating Scale in children receiving postoperative care—a pre-post study

**DOI:** 10.1007/s00431-026-06932-4

**Published:** 2026-04-27

**Authors:** A. Angenent, J. M. Maaskant, L. Nieland, C. L. Hoedjes, A. M. P. Klaassen, S. van Engelen, T. Sieswerda-Hoogendoorn

**Affiliations:** 1 Department of Pediatrics, Emma Children’s Hospital, Amsterdam UMC, Amsterdam, the Netherlands; 2 Amsterdam Reproduction & Development Research Institute, Amsterdam, the Netherlands; 3 Department of Social Pediatrics, Emma Children’s Hospital, Amsterdam UMC, Amsterdam, the Netherlands

**Keywords:** Patient comfort, Pain management, Pain measurement, Comfort assessment, Postoperative care, Pediatrics

## Abstract

This study evaluates the effect of a comfort assessment using focus language compared with pain assessment using the Numeric Rating Scale (NRS) on postoperative length of stay (LOS), analgesic use and patient and parental satisfaction. Prospective pre-post study was conducted on a general pediatric ward. During the pre-intervention period, pain was assessed using the NRS. Post-intervention, comfort was assessed by focus language using comfort scores; an approach that shifts the conversation from the reduction of pain to the improvement of comfort. Eligible participants were children ≥ 6 years receiving postoperative care for > 24 h, able to report a NRS. Primary outcome was LOS (hours). Secondary outcomes were analgesic use (hours) and patient and parental satisfaction. Multivariable linear regression was used to adjust for baseline differences, potential confounding and effect modification. Pre-intervention 93 patients were included and post-intervention 99. The adjusted mean LOS in the post-intervention group: 94 h, was higher than in the pre-intervention group: 74 h (*p* < 0.01). The estimated LOS was significantly higher post-intervention, suggesting more complex surgeries during that period; no statistically significant difference was found between estimated and actual LOS. Analgesic use and patient and parental satisfaction were comparable. *Conclusion*: A focus on comfort during pediatric postoperative care did not statistically significantly impact the LOS, the analgesic use, and patients and parents’ satisfaction. Shifting from pain-focused to comfort-focused communication appears to be a safe, feasible, and promising approach. Future research requires more homogeneous patient groups to determine the impact of using comfort-focused language.

**Table:** 

**What is Known:** • *The NRS is commonly used to assess pain in children ≥ 6 years.* • *Pain-related communication may influence the perception of pain.*	
**What is New:** • *Comfort assessment instead of NRS did not change analgesic use or patient/parent satisfaction.* • *Comfort-focused communication appears safe and feasible in pediatric postoperative care.*

**Supplementary Information:**

The online version contains supplementary material available at 10.1007/s00431-026-06932-4.

## Introduction

The measurement of pain using the Numerical Rating Scale (NRS) is widely used in verbal children aged 6 years and older, yet its validity and potential unintended effects are increasingly debated especially for the measurement of postoperative pain [1, 2]. Although valued for its simplicity, repeatedly asking children to rate their pain may heighten pain perception, fear and functional disability [3–5]. These effects can be amplified by caregivers’ distress and focus on pain, while nonpain-related factors such as depression may also influence NRS scores [6–9].

Pain and distress are significant clinical problems, associated with elevated metabolism, immune suppression, and impaired brain development [10, 11]. In surgical patients, these problems are common and can prolong hospital stay [12–16]. Accurate pain assessment is therefore essential for guiding treatment and evaluating its effectiveness. However, growing evidence highlights the role of communication in shaping pain perception, suggesting that assessment methods themselves may influence outcomes [7, 9, 17].

Focus language is a psychological approach that shifts the conversation from pain and its reduction, to comfort and its improvement [18]. Similar interventions—such as medical hypnosis, breathing techniques, distraction, and positive language—have been shown to reduce pain and distress in children during medical procedures [17–19]. Moreover, in adults, studies have shown that the words used by healthcare providers can modulate analgesic effects and affect patients’ anxiety and pain experience. For example, Bingel et al. [20] showed that when administering a powerful painkiller to healthy volunteers exposed to a painful stimulus, the effect of the medication was influenced by the raised expectation of pain relief. When participants were told they were receiving a very powerful painkiller, they reported less pain compared to those who were told that the drug might not help. Furthermore, a randomized trial conducted by Varelmann et al. [21] showed that women receiving reassuring words about the procedure before administration of the local anesthetic injection, prior to inserting an epidural, reported lower median pain scores compared to the women receiving harsher words. Moreover, Chooi et al. [22] conducted a randomized trial comparing pain and comfort scores in women after caesarean section. Their results showed more discomfort and analgesia use when women were asked to rate pain instead of comfort, suggesting that the wording of assessment scales can influence patients’ pain perception [22].

Despite these promising findings in adults, the effectiveness of focus language in pediatric postoperative care remains unstudied. We designed a study evaluating the differences between comfort assessment using focus language and pain assessment using the NRS, on postoperative length of stay, analgesic use, and patient and parental satisfaction in postoperative children 6–18 years old.

## Materials and methods

### Study design and setting

This prospective pre–post study was conducted at a general pediatric ward of Emma Children’s Hospital, Amsterdam UMC. It comprised three phases: pre-intervention, implementation, and post-intervention. We have chosen for a pre-post study design because we aimed to evaluate the effectiveness of an intervention by comparing health outcomes before and after its implementation. This design is commonly used to evaluate an intervention in a real-world context, when a randomized trial is not feasible.

### Participants

We included children aged 6–18 years, receiving > 24 h postoperative care, able to self-report pain or comfort and fluent in Dutch (patient and/or parents).

### Outcomes

The primary outcome of this study is postoperative length of stay (LOS, hours). The secondary outcomes are the difference between the estimated LOS and the actual LOS (delta LOS, hours), the duration of analgesic use (hours), and patient and parent satisfaction with pain management.

### Procedures

#### Pre-intervention (Sept 2021–July 2022)

Patients assessed pain, reporting the NRS (0 = no pain, 10 = worst pain imaginable) to their nurse every 8 h during the first 72 h post-surgery. Nurses assessed a nurse observed NRS as well, based on their observation of the patient. Both scores were documented in the electronic patient file (EPF). Using the NRS is the standard procedure of pain assessment in the participating ward; no additional training was needed.

#### Implementation (Aug–Sept 2022)

All healthcare professionals of the participating ward involved in the care of the children were trained in focus language. The following healthcare professionals were trained: nurses, physician assistants, surgeons, nurse practitioners, physiotherapists, dietitians, and pain specialists. The training was provided by research team members during clinical lessons and dayshift evaluations, in which the nursing team collectively reviewed the care provided that day. In addition, reminder cards with a decision tree about the use of focus language and how to conduct a comfort score (see Supplementary Figure S1) were handed out to the healthcare professionals, and posters with supportive information were placed in the ward. Nurses were trained to report a patient reported comfort score in the EPF instead of the NRS.

#### Post-intervention (Sept 2022–Oct 2023)

Patients assessed comfort using the comfort score (0 = worst day, 10 = best day) every 8 h during the first 72 h post-surgery. Pain was not actively mentioned by healthcare professionals. If the patient gave a comfort score ≤ 5, causes and supportive interventions were discussed. Pain was only addressed if raised by the patient. Nurses assessed a nurse observed NRS, conform pre-intervention period. The comfort score and the NRS were both documented in the EPF, as part of the new standard procedure.

#### Sample size

Based on mean LOS of the participating ward in 2020 of 4.6 days (110 h) and a 15% reduction (α = 0.05, power = 80%), 108 participants were required per group (nQuery version 8.5.1.).

#### Ethics

The Institutional Review Board of Amsterdam University Medical Center (UMC) confirmed the study was not subject to the Medical Research Involving Human Subjects Act (WMO) (W21_342#21.379). Data were pseudonymized, stored securely, and analyzed in compliance with General Data Protection Regulations. Consent was not required as the study was conducted in compliance with the Dutch law on Quality of Healthcare and evaluated quality of care.

### Data collection

Data were extracted from the EPF by three researchers (Ariana Angenent, Charlotte Hoedjes, Linda Klaassen); a random sample of 10% was independently verified by the head researcher (Tessa Sieswerda-Hoogendoorn).

### Sample characteristics

The following sample characteristics were collected from the EPF: age (years), gender, type of surgery, and medical specialism. We collected the highest and the lowest nurse observed NRS per participant. These scores from the pre-intervention and post-intervention period were used to explore the pain levels of the patients during both periods, in order to assess whether the groups were comparable. Additionally, we collected the highest and the lowest patient reported NRS per participant (pre-intervention) and the highest and lowest comfort scores per participant (post-intervention).

### Outcome measures

#### Length of postoperative stay (LOS)

The LOS (hours) was defined as the time the patient arrived at the Post Anesthesia Care Unit (PACU) until the time that the patient was discharged from the hospital. When the time of arriving at the PACU was not registered in the EPF, the time of departing the operating room was used to note a LOS as accurate as possible.

#### Delta LOS

Additional to the *actual LOS*, the *delta LOS* (hours) was defined as: the difference between the expected length of postoperative stay and the actual length of postoperative stay. The *expected LOS* was estimated pre-hospitalization and registered in the EPF.

#### Analgesics use

The duration of analgesic use (in hours) for every type of analgesic was measured from the moment the participants arrived at the PACU until the last gift of the analgesic, or until the moment of discharge. The type of analgesics was noted, as well as the method of medication administration. The duration of analgesics use was reported for different subgroups: parenteral analgesics, enteral analgesics, parenteral opioids, and enteral opioids.

#### Satisfaction

The Netherlands Federation of University Medical Centers (NFU) survey was used to explore the satisfaction of the patients and their parents or caregivers about the postoperative pain management. The NFU survey is a national survey which is sent electronically to every patient or their parent who has been hospitalized in a Dutch University Medical Centre to monitor the experiences of patients and parents [23]. The survey includes a question regarding pain management, addressing the patients (aged 8–17 years) and the parents (patients aged 0–8 years).

#### Confounders and effect modifiers

The following potential confounders were collected: (post-)surgery complications, (registered) psychiatric problems, and the presence of a chronic condition associated with chronic pain. We used the following definitions:

- (Post-)surgery complications: (internal) bleeding, (wound) infection, urinary tract infection, anastomotic leakage after bowel surgery, postoperative ileus, pneumonia, and any situation that needed a re-intervention.
- Psychiatric problems: mental health problems which daily affects the way a person thinks, feels and behave, e.g., anxiety disorder, depression, posttraumatic stress disorder, attention deficit hyperactivity disorder, and autism spectrum disorder.
- Chronic conditions associated with chronic pain, e.g., inflammatory bowel disease, therapy resistant constipation, medically unexplained symptoms, hereditary motor and sensory neuropathy, and arthritis as these conditions may change the pain experience and management [5, 12, 24].

Patients with (post-)surgery complications, tend to need longer postoperative care due to the complication. This may affect the primary outcome. Regarding patients with psychiatric problems and patients with chronic conditions associated with chronic pain, it is clinically plausible these subgroups were affected differently by the focus language as these patients often need specific treatment during postoperative care [5, 12, 24]. Regarding the use of analgesics, we considered LOS an effect modifier. When the LOS is longer, it is plausible the duration of analgesic use is longer as well. Additional subgroup analyses are reported in Supplementary Table S1.

### Statistical analysis

Descriptive statistics of continuous values were expressed as mean and standard deviation (SD). If not normally distributed, median and interquartile range (IQR) were used. Baseline differences between the pre- and post-intervention groups were explored using the Fisher exact test (dichotomous data), the chi-square test or the Wilcoxon-rank sum test (categorical data), or the unpaired *T*-test (continuous data). The assumptions (normal distribution and homoscedasticity) were tested using the Shapiro-Wilkinson test, the Levene’s test and by visual inspection of the histograms, QQ-plots and boxplots of the data. If the data were not normally distributed, the variable was log-transformed. If the data did not have homoscedasticity, the Yates correction was considered for the unpaired *T*-test. The Wilcoxon-rank sum test was used in case both assumptions were not met. We considered *p* < 0.05 statistically significant. All analyses were performed in R Studio (v4.0.4).

### Missing data

Only complete cases were included. For complications, psychiatric problems, and chronic conditions, absence in the EPF was interpreted as not present. NFU survey data were analyzed as available; missingness could not be assessed.

## Results

### Baseline characteristics of the participants

**Fig. 1:**
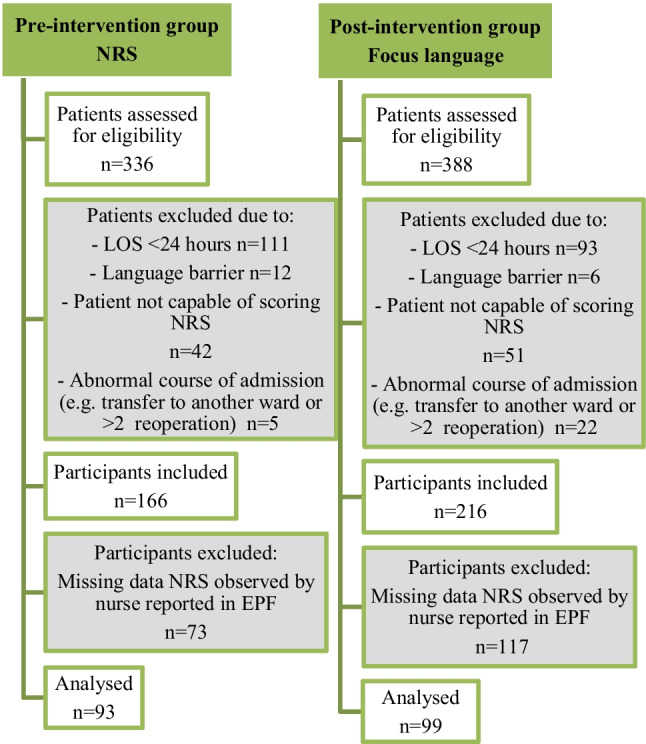
Flow diagram of the participants in the pre- and post-intervention group

In total, 724 patients were assessed for eligibility; of which 315 patients were not included due to not meeting inclusion criteria, 27 patients were excluded due to abnormal course of admission and 190 were excluded due to missing data (Fig. 1). Resulting in an inclusion of 93 patients during the pre-intervention period and 99 patients during the post-intervention period (Fig. 1). The following sample characteristics showed no statistically significant differences between the pre- and post-intervention periods: age, gender and type of medical specialism (Table 1).

**Table 1:** Sample characteristics

Characteristics	Pre-intervention group (NRS)	Post-intervention group (focus language)	*p*-value^a^
**Participants, *n***	93	99	-
**Age (in years)**			0.82^b^
Median [IQR^#^]	13.16 [11.00; 16.00]	13.42 [12.00; 15.00]	
**Gender**			0.47^c^
Male, *n* (%)	56 (60.2)	54 (54.5)	
Female, *n* (%)	37 (39.8)	45 (45.5)	
**Type of medical specialism**			0.08^c^
General pediatric surgery, *n* (%)	47 (50.5)	46 (46.5)	
Pediatric plastic surgery, *n* (%)	7 (7.5)	9 (9.1)	
Pediatric orthopedic surgery, *n* (%)	23 (24.7)	39 (39.4)	
Pediatric pulmonology, *n* (%)	3 (3.2)	-	
Trauma surgery, *n* (%)	4 (4.3)	3 (3.0)	
Pediatric gastroenterology, *n* (%)	1 (1.1)	-	
General pediatrics, *n* (%)	1 (1.1)	-	
Pediatric urology, *n* (%)	4 (4.3)	1 (1.0)	
Pediatric oral and maxillofacial surgery, *n* (%)	2 (2.2)	-	
Neurosurgery, *n* (%)	1 (1.1)	-	
Otorhinolaryngology, *n* (%)	-	1 (1.0)	
**Types of surgery, *n***	68	71	< *0.01*^*c*^
**Complications**			0.24^c^
No, *n* (%)	86 (92.5)	86 (86.9)	
Yes, *n* (%)	7 (7.5)	13 (13.1)	
**Confounders and effect modifiers**			
Diagnosed psychiatric problems OR chronic conditions associated with chronic pain, *n* (%)	15 (16.1)	24 (24.2)	0.28^c^
Diagnoses chronic conditions associated with chronic pain, *n* (%)	14 (15.1)	23 (23.2)	
Diagnosed psychiatric problems, *n* (%)	2 (2.2)	2 (2.0)	
**Comfort and pain assessment**			
Highest NRS by nurse, median [IQR^#^]	4 [3; 6]	4 [3; 5]	0.94^b^
Lowest NRS by nurse, median [IQR^#^]	0 [0: 1]	1 [0; 2]	< *0.01*^b^
Highest NRS by patient, median [IQR^#^]	6 [4; 7]	-	
Lowest NRS by patient, median [IQR^#^]	0 [0; 1]	-	
Lowest comfort score by patient, median [IQR^#^]	-	5 [4; 6]	
Highest comfort score by patient, median [IQR^#^]	-	8 [7.25; 9]	

^#^IQR = Interquartile range

^a^A *p*-value < 0.05 was considered statistically significant

^b^Wilcoxon-rank sum test

^c^Fisher exact test

### Length of postoperative stay (LOS)

The mean *LOS* was statistically significant longer post-intervention 115.9 h (SD 10.98) compared to pre-intervention 84.3 h (SD 10.35; expβ = 1.38, *p* < 0.01). After adjustment for confounding, this remained statistically significant (mean 94.3 h vs. 74.1 h; expβ = 1.27, *p* < 0.01;). See Table 2.

**Table 2:** Results of multivariable linear regression analyses for postoperative length of stay (LOS)

Primary outcome	Pre-intervention group (*n* = 93)			Post-intervention group (*n* = 99)			Exp(β-Coeff)^a^	*p*-value^b^
	Mean	SD^*^	Exp(SE^○^)^a^	Mean	SD^*^	Exp(SE^○^)^a^		
Length of postoperative hospital stay (in hours) without adjustment for confounders	84.28	10.35	1.07	115.92	10.98	1.10	1.38	< *0.01*
Length of postoperative hospital stay (in hours) with adjustment for confounders^c^	74.13	10.31	1.07	94.29	10.89	1.09	1.27	< *0.01*

^*^SD = Standard Deviation, ^○^SE = Standard Error

^a^The dependent variable was log-transformed to meet the assumptions of the linear regression

^b^A *p*-value < 0.05 was considered statistically significant

^c^Corrected for the variable: psychiatry or chronic pain and complications

### Difference between estimated and actual LOS

The mean *delta LOS* was not statistically significant different for patients in the post-intervention group: 42.95 h (SD 10.82) compared to the pre-intervention group: 37.78 h (SD 10.31, expβ = 1.04, *p* = 0.64). After adjustment for confounding, this remained not statistically significant (mean 23.60 h vs. 26.20 h; expβ = 0.97, *p* = 0.77). See Table 3. To perform the delta LOS analyses, two participants in the post-intervention group were excluded due to missing preoperative estimated LOS in the EPF.

**Table 3:** Results of additional multivariable linear regression analyses for delta LOS

Primary Outcome	Pre-intervention group (*n* = 93)			Post-intervention group (*n* = 97)			Exp(β-Coeff)^a^	*p*-value^b^
	Mean	SD^*^	Exp(SE^○^)^a^	Mean	SD^*^	Exp(SE^○^)^a^		
Delta length of postoperative hospital stay (in hours) without adjustment for confounders	37.78	10.31	1.07	42.95	10.82	1.10	1.04	0.64
Delta length of postoperative hospital stay (in hours) with adjustment for confounders^c^	26.20	10.29	1.07	23.60	10.77	1.09	0.97	0.77

^*^SD = Standard Deviation, ^○^SE = Standard Error

^a^The dependent variable was log-transformed using (log(y + α)) to meet the assumptions of the linear regression

^b^A *p*-value < 0.05 was considered statistically significant

^c^Corrected for the variable: psychiatry or chronic pain and complications

### Analgesic use

We adjusted the following analyses for effect modification. The mean duration of *parenteral analgesics* use (opioids and non-opioids) was not statistically significant different for patients in the post-intervention group compared to the pre-intervention group: 18.94 h (SD 12.03) versus 20.99 h (SD 11.52), *p* = 0.59. The mean duration of *enteral analgesics* use (opioids and non-opioids) was not statistically significant different for the patients in the post-intervention group compared to the pre-intervention group: 51.58 h (SD 11.56) versus 38.61 h (SD 11.10), *p* = 0.05. The mean duration of *parenteral opioids* use for the patients in the post-intervention group remained not statistically significant different from the pre-intervention group: 1.13 h (SD 13.09) versus 1.01 h (SD 14.63), *p* = 0.67. The mean duration of *enteral opioids* use for the patients in the post-intervention group was not statistically significant different from the pre-intervention group: 10.87 h (SD 14.65) versus 5.79 h (SD 18.51), *p* = 0.11. See Supplementary Table S2.

### Satisfaction

NFU surveys were completed by 16 patients and 61 parents’ pre-intervention, and 18 patients and 89 parents’ post-intervention. Satisfaction scores regarding pain management were not statistically significant different. *Patient satisfaction*: median 3 [IQR 3–3] in pre- and post-intervention period (*p* = 0.56). *Parental satisfaction*: median 3 [IQR 3–3] in pre- and post-intervention period (*p* = 0.06). See Supplementary Table S3.

### Post-hoc analyses

A statistically significant difference in surgery type was observed between pre- and post-intervention groups. A total of 130 types of surgeries were performed during this study, of which 9 types of surgeries were performed in both the pre- and post-intervention period. See Supplementary Table S4 for an overview of the most commonly performed operations in the pre- and post-intervention group.

## Discussion

### Main findings

Contrary to expectations, we found that LOS was longer when comfort assessment was done with focus language instead of with the NRS. Furthermore, we found that analgesic use and patient/parental satisfaction in children receiving postoperative care were not influenced by the use of focus language.

This study illuminates that LOS may not be the right outcome measure to investigate the effect of focus language. First of all, the longer LOS in the intervention group may reflect variations in surgery types between the pre- and post-intervention groups rather than the intervention’s effect. During the pre-intervention period, the COVID-19 pandemic affected scheduling, which caused catch-up surgeries during the post-intervention period, likely increasing heterogeneity in surgery types [25]. Since LOS is influenced by surgery type, this complicates the interpretation of our results [26]. The estimated LOS was significantly higher post-intervention, suggesting that more complex surgeries requiring longer LOS were performed during that period. To address the surgical heterogeneity, we focused on the difference between estimated and actual LOS (Delta LOS) additional to the primary outcome. As Delta LOS did not statistically significantly differ between groups, this suggests the intervention did not adversely affect LOS.

Second, LOS has dramatically decreased in the period 2005–2012, but has been stable the past 13 years [27]. For children 0–19 years the LOS has been approximately 4 days since then [27]. Given that clinical care has been tailored to meet discharge criteria in the most efficient way possible, there may be a ceiling effect with limited opportunity for further reduction.

Although we did not find an effect of focus language on reduction of LOS, the intervention itself might reduce stress and enhance comfort. This was shared by anesthesia staff providing the post-operative pain management on the pediatric wards. The staff were so pleased with the new approach that it was implemented throughout the hospital before the results of this study were available [28]. Recently, Van Dorp et al. [29] and Edwards et al. [30] found that in adults visiting the emergency department because of pain, pain scores and comfort scores did not significantly differ. Therefore, pain and comfort scores might be interchangeable, supporting the safety of studying the effect of cognitive reframing and the use of comfort scores.

This positive attitude towards the new approach is supported by the finding that patients and parents remained equally satisfied about pain management, although they were never questioned about pain [30, 31]. Furthermore, patients received the same amount of painkillers in the intervention group, suggesting that patients’ needs were still adequately recognized and treated. Inconsistent with our results were the findings of Chooi et al. [22] regarding their study among women receiving postoperative care after a caesarean section. They found that women in the comfort group requested even less painkillers compared to women in the standard NRS group. They also found that the negative suggestions used in the NRS group seem to have resulted in an increased number of patients reporting that their post-operative sensations were unpleasant, more bothersome, and perceived as tissue damage and injury rather than healing and recovery as mentioned in het comfort group Chooi et al. [22]. The difference with our findings is probably caused by the fact that their patients all underwent the exact same type of surgery, which commonly has a quite similar postoperative course. This makes their groups very homogeneous and their results not influenced by factors other than the intervention.

### Limitations

This study has several limitations. As mentioned above, when selecting the before-after design, we did not anticipate heterogeneity in surgery types, given the duration of inclusion during pre- and post-intervention period (11–14 months) and the homogeneity of the specialisms. The heterogeneity in surgery types may explain the difference in LOS rather than the effect of focus language. Secondly, the sample size was smaller than originally calculated due to missing data. The before–after design, the heterogeneity in surgical procedures, and the smaller-than-planned sample size, may limit the interpretability of the observed effect.

Furthermore, the uncertain fidelity of the focus language implementation may have affected internal validity. At the end of the implementation period of this research, no measurements were conducted regarding how well the focus language was used during practical care. This limitation results in uncertainty about protocol adherence; whether the healthcare professionals used the focus language as described. This uncertainty also introduces the potential for contamination bias. Because patients in the intervention group may have occasionally received elements of standard care, and patients in the control group may have been inadvertently exposed to components of the intervention, the distinction between groups may have been reduced. Such contamination can dilute the true effect of the intervention, resulting in an underestimation of the results.

Additionally, the relatively low response rate to the NFU survey (25%) lacks power and therefore can only be seen as an exploration of the patient/parental satisfaction. Finally, the study was conducted in a single center, which may limit the generalizability of the findings.

### Future research

Future studies should use homogeneous surgical populations and consider outcomes more directly related to pain and distress rather than LOS, as this may provide a clearer understanding of the intervention’s true effect. As we did not administer any (additional) patient questionnaires, our insight into the patient experience is limited. Administering questionnaires and conducting interviews—such as measures of patient satisfaction, perceived control, or procedural anxiety—could provide more in-depth insight into outcomes that are relevant from the patient’s perspective. Subgroups (e.g., children with psychiatric or chronic pain conditions, or different age groups) may respond differently to focus language, as our exploratory analyses suggested shorter LOS in patients with chronic pain or psychiatric conditions.

## Conclusion

Shifting from pain-focused to comfort-focused communication may be a safe and feasible approach in pediatric postoperative care. Patients and parents remain satisfied, analgesic use is unaffected, and the method shows promise for enhancing the patient experience. Future research should target homogeneous populations and explore the perspectives of both patients and healthcare professionals to maximize benefits.

## Supplementary Information

Below is the link to the electronic supplementary material.

ESM 1 Supplementary Material 1 (DOCX 58.8 KB)

## Data Availability

The data that support the findings of this study are available from the corresponding author upon reasonable request, in accordance with FAIR data principles (Findable, Accessible, Interoperable, and Reusable).

## Abbreviations

EPF: Electronic patient file
IQR: Interquartile range
LOS: Postoperative length of hospital stay
NFU: Netherlands Federation of University Medical Centers
NRS: Numeric Rating Scale
PACU: Post Anesthesia Care Unit
SD: Standard deviation
UMC: University Medical Center
VATS: Video-assisted thoracoscopic surgery
WMO: Medical Research Involving Human Subjects Act
