# Supplementary material for: Focus on comfort: the effect of focus language on postoperative hospital stay compared to the Numeric Rating Scale in children receiving postoperative care—a pre-post study
Source: Eur J Pediatr. 2026 Apr 27;185(5):306. doi: 10.1007/s00431-026-06932-4 (PMC13121221; doi:10.1007/s00431-026-06932-4)
Supplement: Supplementary file 1 — Supplementary Material 1 (DOCX 58.8 KB) [file 431_2026_6932_MOESM1_ESM.docx]

**Focus on Comfort - The effect of focus language on postoperative hospital stay compared to the Numeric Rating School in children receiving postoperative care – A pre-post study**

Authors:

Angenent A.1,

Maaskant J.M., PhD 1,2

Nieland L.1,

Hoedjes C.L.1,

Klaassen A.M.P.1,

Van Engelen S.1,

Sieswerda-Hoogendoorn T., MD, PhD 3.

1 Department of Pediatrics, Emma Children’s Hospital, Amsterdam UMC, Amsterdam,

the Netherlands.

2 Amsterdam Reproduction & Development Research Institute, Amsterdam, the Netherlands.

3 Department of Social Pediatrics, Emma Children’s Hospital, Amsterdam UMC,

Amsterdam, the Netherlands.

Corresponding author: T. (Tessa) Sieswerda-Hoogendoorn, [t.sieswerda@amsterdamumc.nl](mailto:t.sieswerda@amsterdamumc.nl)

**Supplementary Figure S1 - Decision tree - Comfort assessment using focus language**

Supporting decision tree for healthcare professionals in clinical practice during the Focus on Comfort study to perform comfort assessment using focus language.

**Supplementary Table S1 - Additional subgroup analyses**

Additional statistical analyses were performed to explore whether focus language affected subgroups differently regarding LOS. The subgroups explored: patients with psychiatric problems or chronic conditions associated with chronic pain, patients without psychiatric problems or chronic conditions associated with chronic pain, patients aged 6-11 years, patients aged 12-18 years, patients with complications and patients without complications.

|  | **Pre-intervention group - NRS** | | | **Post-intervention group - Focus language** | | |  |  |
| --- | --- | --- | --- | --- | --- | --- | --- | --- |
|  | N (%) | Median | IQR^#^ | N (%) | Median | IQR^#^ | W-value^1^ | P-value^2^ |
| LOS for patients with psychiatric problems or a chronic condition  (in hours) | 15  (16.13) | 172.0 | [150.0; 193.0] | 24  (24.24) | 157.0 | [112.5; 222.5] | 180.5 | 1 |
| LOS for patients without psychiatric problems or a chronic condition  (in hours) | 78  (83.87) | 78.50 | [47.25; 114.25] | 75  (75.76) | 100.0 | [69.5; 166.5] | 2139.5 | *<0.01* |
| LOS for patients aged  6-11 years  (in hours) | 29  (31.18) | 95.0 | [50.0; 169.0] | 20  (20.20) | 75.5 | [67.5; 117.8] | 298.5 | 0.87 |
| LOS for patients aged 12 years and older  (in hours) | 64  (68.82) | 82.0 | [48.5; 144.2] | 79  (79.80) | 141.0 | [73; 154.2] | 1717.5 | *<0.01* |
| LOS for patients with complications (in hours) | 7  (7.53) | 167.0 | [107.5; 248.5] | 13  (13.13) | 257.0 | [213.0; 389.0] | 26 | 0.13 |
| LOS for patients without complications (in hours) | 86  (92.47) | 90.50 | [48.25; 142.75] | 86  (86.87) | 107.5 | [70.0; 164.0] | 2947.5 | 0.02 |

^#^IQR = Interquartile range

^1^ W-value of the Wilcoxon Rank sum test

^2^ A p-value <0.05 was considered statistically significant.

**Supplementary Table S2 – Results of multivariable linear regression analyses for analgesic use**

|  | **Pre-intervention group (n=93)** | | | **Post-intervention group (n=99)** | | |  |  |
| --- | --- | --- | --- | --- | --- | --- | --- | --- |
| **Secondary Outcomes** | Mean | SD^*^ | Exp(SE^○^)^1^ | Mean | SD^*^ | Exp(SE^○^)^1^ | Exp(β-Coeff)^1^ | P-value^2^ |
| Duration of parenteral analgesic use (in hours) without adjustment for confounders or effect modifier | 46.92 | 11.20 | 1.16 | 56.74 | 12.25 | 1.23 | 1.21 | 0.36 |
| Duration of parenteral analgesic use (in hours) with adjustment for confounders and effect modifier^3^ | 20.99 | 11.52 | 1.19 | 18.94 | 12.03 | 1.21 | 0.90 | 0.59 |
| Duration of enteral analgesic use (in hours) without adjustment for effect modifier | 78.13 | 10.88 | 1.13 | 132.25 | 11.76 | 1.18 | 1.69 | *<0.01* |
| Duration of enteral analgesic use (in hours) with adjustment for effect modifier^4^ | 38.61 | 11.10 | 1.15 | 51.58 | 11.56 | 1.16 | 1.34 | 0.05 |
| Duration of parenteral opioid use (in hours) without adjustment for confounders or effect modifier | 7.14 | 11.82 | 1.23 | 12.11 | 13.31 | 1.34 | 1.70 | 0.07 |
| Duration of parenteral opioid use (in hours) with adjustment for confounders and effect modifier^3^ | 1.01 | 14.63 | 1.52 | 1.13 | 13.09 | 1.32 | 1.12 | 0.67 |
| Duration of enteral opioid use (in hours) without adjustment for effect modifier | 11.32 | 12.84 | 1.33 | 23.89 | 14.46 | 1.45 | 2.11 | 0.05 |
| Duration of enteral opioid use (in hours) with adjustment for effect modifier^4^ | 5.79 | 18.51 | 1.92 | 10.87 | 14.65 | 1.47 | 1.88 | 0.11 |

*SD = Standard Deviation, ^○^SE = Standard Error

^1^ The dependent variable was log-transformed to meet the assumptions of the linear regression.

^2^ A p-value <0.05 was considered statistically significant.

^3^ Corrected for the variables: “psychiatry or chronic pain”, “complications” and “length of postoperative stay”. Restricted cubic splines with 3 knots was applied on the variable “length of postoperative stay”.

^4^ Corrected for the variable: “length of postoperative stay”. Restricted cubic splines with 3 knots was applied.

**Supplementary Table S3 - Results non-parametric test analysis for patient and parental satisfaction**

|  | **Pre-intervention group** | | | **Post-intervention group** | | |  |  |
| --- | --- | --- | --- | --- | --- | --- | --- | --- |
| **Secondary Outcomes** | N | Median | IQR^#^ | N | Median | IQR^#^ | W-value^1^ | P-value^2^ |
| Patient satisfaction – NFU Survey | 16 | 3 | [3; 3] | 18 | 3 | [3; 3] | 127.5 | 0.56 |
| Parental satisfaction – NFU Survey | 61 | 3 | [3; 3] | 89 | 3 | [3; 3] | 2995 | 0.06 |

^#^IQR = Interquartile range.

^1^ W-value of the Wilcoxon Rank sum test.

^2^ A p-value <0.05 was considered statistically significant.

**Supplementary Table S4 – Supplementary results regarding types of surgical procedures per group**

| **Types of surgical procedures** | **Pre-intervention**  **(n)** | **Mean LOS* (hours)** | **Post-intervention**  **(n)** | **Mean LOS* (hours)** |
| --- | --- | --- | --- | --- |
| Cholecystectomy | 4 | 36.25 | 2 | 46.5 |
| Modified Dunn procedure | - | - | 4 | 127.75 |
| Nuss bar procedure | 12 | 89.33 | 4 | 83 |
| Re-spondylodesis | - | - | 8 | 160.88 |
| Subtotal colectomy | 2 | 168.5 | 4 | 202 |
| Video-assisted thoracoscopic surgery (VATS) | 3 | 146 | 1 | 219 |
| Various surgical procedures performed 1 or 2 times | 72 | 109.7 | 76 | 144.1 |

*Mean postoperative length of stay (LOS) without adjustment for confounding.
